# The relationship between stress and health-related quality of life and the mediating role of self-efficacy in Norwegian adolescents: a cross-sectional study

**DOI:** 10.1186/s12955-022-02075-w

**Published:** 2022-12-09

**Authors:** Erik Grasaas, Siv Skarstein, Hilde Timenes Mikkelsen, Milada Cvancarova Småstuen, Gudrun Rohde, Sølvi Helseth, Kristin Haraldstad

**Affiliations:** 1 Department of Health and Nursing Science, Faculty of Health and Sport Sciences, University in Agder, Post Box 422, 4604 Kristiansand, Norway; 2 Department of Nursing and Health Promotion, Faculty of Health Sciences, Oslo Metropolitan University, Oslo, Norway; 3 Department of Clinical Research, Sorlandet Hospital, Kristiansand, Norway

**Keywords:** Adolescents, Health-related quality of life, Stress, Self-efficacy, Mediation

## Abstract

**Background:**

During the transitional phase from childhood to adulthood, adolescents encounter many changes and challenges. Stress is associated with reduced health-related quality of life (HRQOL) in adolescents and, thus, impacts all aspects of their life. Adolescents’ thoughts and beliefs in their capacity may be essential with regard to their subjective perception of stress and coping with it. Insights into the complexity of stress and exploration of the possible underlying mechanisms in adolescence are needed. We sought to describe stress, HRQOL, and self-efficacy and explore the association between stress and HRQOL by testing for self-efficacy as a possible mediator in adolescents.

**Methods:**

In total, 696 school-based adolescents aged 14–15 years participated in this study. Participants were recruited from 22 schools in the Eastern and Southern parts of Norway. All participants completed an electronic survey in their respective classrooms. The survey included demographic data, the Perceived Stress Questionnaire, the KIDSCREEN-27 questionnaire measuring HRQOL, and the General Perceived Self-Efficacy Scale. Statistical analyses were conducted using the PROCESS macro for SPSS Statistics software by Andrew Hayes model 4.

**Results:**

Descriptive analyses revealed overall low levels of stress with a score of 0.29 (SD, 0.15). Nevertheless, stress was negatively associated with all HRQOL subscales: physical well-being (B =  − 25.60), psychological well-being (B =  − 38.43), autonomy and parents (B =  − 28.84), social support and peers (B =  − 21.05), and school environment (B =  − 30.28). Furthermore, these respective associations were all mediated by self-efficacy, which explained approximately one-fifth of the reduction in HRQOL. The highest degree of mediation and, thus, the largest indirect effect was estimated for the HRQOL subscale physical well-being (31.7%).

**Conclusions:**

Our findings extend prior research on the mechanisms underlying the relationship between perceived stress and HRQOL in adolescents. They demonstrated that perceived stress explained most of the reduction in the HRQOL after adjusting for the effect of self-efficacy. Hence, stress itself appears to be an important target for future interventions to enhance HRQOL, rather than purely focusing on increasing self-efficacy to enhance the HRQOL in adolescents. Our findings highlight the importance of a better understanding of the underlying mechanisms to develop strategic and accurate interventions for adolescents.

## Background

During the transitional phase from childhood to adulthood, adolescents may encounter many stressful changes and challenges concomitantly. The drive for independence and greater autonomy, brain development, pressure to conform to peers, exploration of sexual identity, physical development, and increased access to and use of technology have been suggested as important factors in this time period [1, 2]. Moreover, perceived stress in adolescence may be related to different expectations and general high demands of being successful in many aspects of life, such as school, social media, and peer relations [3]. Hence, adolescence is often defined as a period of heightened stress [4]. According to Lazarus and Folkman, stress is defined as a relationship between the person and their environment that is appraised by the person as taxing or exceeding their resources and as endangering their well-being [5]. Previous stress experiences may predict the stress response and coping in adolescents. The rate of perceived stress experiences among adolescents in modern society is reported to be high (up to 50%) [6], and the stress levels among adolescents are even higher than those in adults [7]. Furthermore, perceived stress and maladaptive coping are reported to be positively associated with adjustment problems in adolescence [8].

According to the World Health Organization’s Mental Health Action Plan, coping with the normal stressors of life enables adolescents to reach their potential [9]. However, the Action Plan highlights that some adolescents have a greater risk of stressful health conditions because of their living conditions, discrimination, stigma, or a lack of access to quality support and services [9]. Norwegian adolescents are likely to possess advantageous prerequisites in terms of living conditions and access to quality support and services compared with adolescents living in other countries. However, the Ungdata, which is a Norwegian national data collection scheme targeted at the youth, showed that approximately half of the Norwegian adolescents in high schools reported having concerns such as “everything feels like a struggle” or feeling like they were “worrying too much” [6]. The survey was conducted almost every year among Norwegian adolescents aged 14–19 years and has since 2010 shown an increase in perceived stress, especially among girls [6]. For instance, two-thirds of the Norwegian girls and one-third of the Norwegian boys reported being often or very often stressed by schoolwork [10].

Given that stress has an impact on healthy behaviors and may influence all aspects of life, the concept of health-related quality of life (HRQOL) is of relevance because it is defined as a multidimensional concept, which is used to assess the adolescents’ subjective well-being in terms of physical, psychological, social, and spiritual aspects of life [11]. The multidimensionality of HRQOL can provide important information about the impact of stress on different aspects of life, and it may serve as a framework for identifying and developing strategies to improve HRQOL [12]. Several studies have reported that stress symptoms are associated with a low HRQOL [13–15]. Nevertheless, a complex situation involving several factors and strong correlations between the HRQOL dimensions and interacting psychological health variables exists [16], which indicates the need to further explore these associations in school-based populations of adolescents.

The determinants of stress include not only social, cultural, and economic factors but also individual attributes, such as the ability to manage own thoughts and emotions [17]. Thus, adolescents’ thoughts and cognition may be essential in terms of the subjective perception of stress. According to Albert Bandura, self-efficacy might act as a mediator between stress experiences and outcomes, such as well-being [18]. A mediator seeks to identify causal-based mechanisms underlying the observed associations of an exploratory nature [19]. A well-known potential mediator is self-efficacy. Because self-efficacy refers to a person’s thoughts and cognition, it may act as a third interacting variable between the observed associations [18]. Bandura defined self-efficacy as “one’s beliefs (cognition) in one’s capability to organize and execute the courses of action required to achieve given results” [20, 21]. General self-efficacy (GSE) has been shown to positively impact HRQOL by reducing stress, and therefore, it increases the HRQOL in adult patients [22, 23]. Moreover, higher degrees of self-efficacy are shown to be related to higher HRQOL scores in adolescents [24]. Self-efficacy was recently revealed as a mediator in the relationship between pain and HRQOL in a school-based sample of Norwegian adolescents [25]. Although several factors may influence the HRQOL in adolescence, highlighting stress as a potential exponential cause and testing for self-efficacy as a mediator appears to be highly relevant for understanding the underlying mechanisms in adolescence.

Thus, the purpose of this study was to describe stress, HRQOL, and GSE in a school-based population of Norwegian adolescents and explore the possible associations between stress and HRQOL by testing GSE as a possible mediator. We hypothesized that stress is negatively associated with HRQOL and that self-efficacy would play a role as a mediator for the associations between stress and HRQOL.

## Methods

### Design

This cross-sectional study was a part of the “Start Young—quality of life and pain in generations” study, which is a longitudinal study aimed at acquiring new knowledge about HRQOL and pain in adolescents and their parents [26]. The present study used data collected at baseline from November 2018 to April 2019.

### Study setting

A total of 59 elementary schools from the south-eastern part of Norway were invited to participate. Twenty-two schools agreed to participate. The schools varied in size and localization (from city to suburb), admitting adolescents with different sociocultural and economic backgrounds. The potential study participants consisted of 1663 adolescents in the 9^th^ grade from the participating schools, of whom 696 finally participated in the study (response rate, 41.8%). The response rate varied across schools from 92.1 to 8.6%.

### Study procedures

Project members visited each school approximately 1 week before the data collection to provide the adolescents with verbal and written information about the study. Written information was also distributed to the parents. Informed consent was obtained from both adolescents and their parents. A web-based questionnaire was administered and completed in the classrooms during school hours. The schools provided the adolescents with computers to complete the questionnaire. One or two project members and a teacher were present to provide assistance when needed. The collected data was stored in a safe data server.

The “Start Young” study was reviewed by the Norwegian Centre for Research Data (Reference Number: 60981), and the necessary approvals were obtained.

## Measures

### Demographic variables

The baseline questionnaire included questions regarding demographic data, including gender, age, parental status, living conditions, and ethnicity. Parental status and living conditions were used as indicators of the family resources and environment. Furthermore, the baseline questionnaire included the following study variables: stress (independent variable), HRQOL (dependent variable), and GSE (mediator variable).

### Questionnaires

#### HRQOL

To assess HRQOL, the Norwegian version of the KIDSCREEN-27 questionnaire was used [27]. This questionnaire is considered a valid and reliable multidimensional measure of HRQOL in adolescents and is organized into the following five subscales: (1) physical well-being, (2) psychological well-being, (3) autonomy and parents, (4) social support and peers, and (5) school environment [28–31]. The KIDSCREEN instrument comprises a 5-point Likert scale (e.g., from “never” to “always”). Each subscale was transformed into Rasch-scores and had *t*-values with a mean of 50 and a standard deviation (SD) of 10 [32]. Higher values in the respective subscales indicate better HRQOL and well-being.

#### Self-efficacy

To assess self-efficacy, the Norwegian 10-item version of the General Perceived Self-Efficacy Scale revised and translated by Røysamb and colleagues was used [33]. This scale is considered a valid and reliable psychometric scale developed to identify a person’s optimistic self-belief and global confidence in one’s abilities across a wide range of challenging situations [34, 35]. It comprises 10 statements that the respondent rates on a scale from 1 (completely wrong) to 4 (completely right). The individual scores are then summed into a total score, wherein higher scores indicate higher levels of GSE.

#### Stress

To assess stress, the Norwegian version of the Perceived Stress Questionnaire (PSQ) was used [36–38]. The PSQ comprises 30 items referring to the last 4 weeks and can be answered with a 4-point rating scale ranging from 1 (“almost never”) to 4 (“almost always”). The Norwegian version of the instrument has been shown to have good reliability and validity [38, 39]. Higher values indicate higher levels of perceived stress. The resulting PSQ total score is linearly transformed between 0 and 1; PSQ = (raw value − 30)/90 [36]. Commonly used cutoff levels of stress with respect to the PSQ are low, < 0.33; medium, 0.33–0.45; moderate, 0.45–0.60; and severe, > 0.60 [36, 37].

### Statistical analyses

**Fig. 1:**
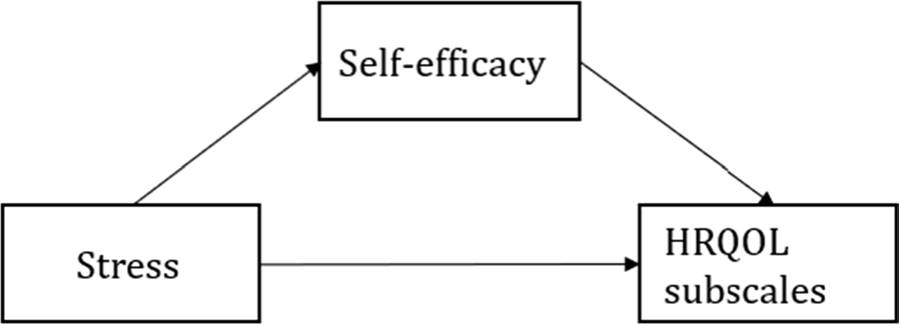
Illustration of the simple mediation model

The statistical analyses were conducted using IBM SPSS Statistics for Windows, Version 25.0 (IBM Corp., Armonk, NY, USA). Demographic data were described using descriptive measures. Continuous variables were described using mean and standard deviation, and categorical variables with frequencies and percentages. Linear regression analyses were conducted between the independent variable stress and dependent variables HRQOL and GSE. Mediation analysis was conducted using the PROCESS macro model 4 for SPSS by Andrew Hayes [19] and the model was controlled for gender, ethnicity, parental status, and living conditions. To increase precision, all the estimates were calculated using bootstrapping. The mediation effect was regarded as statistically significant if the 95% confidence interval (CI) for this effect did not include 0. Finally, both the indirect and direct effects were each divided by the total effect, multiplied by 100 and, thus, presented as percentages. *p*-values < 0.05 were considered statistically significant and all tests were two-sided. We used a simple mediation model presented in Fig. 1.

## Results

### Participants

In total, 696 school-based adolescents (more girls [57.5%] than boys [42.5%]) participated in this study. The participants’ ages were 13 years (1.6%), 14 years (88.2%), and 15 years (10.2%). Most adolescents (79.1%) had parents who were born in Norway, 12.5% of the adolescents had one parent born in Norway, and 8.3% had both parents born in other countries.

### Descriptive data of study variables: stress, HRQOL, and GSE

The mean level of stress for the total study sample was categorized as low with a score of 0.29 (SD, 0.15). Girls reported a higher mean stress level (0.33 [SD, 0.16]), which lies on the cutoff level between low and medium levels, than boys (0.24 [SD, 0.14]). The study sample had a mean GSE of 31.08 (SD, 4.31). Girls had a lower mean GSE (30.25 [SD, 4.27]) than boys (32.21 [SD, 1.9]). Boys had higher scores in the HRQOL subscales than girls (Table 1). The largest gender difference in mean was observed for the HRQOL subscale psychological well-being, wherein girls and boys had a mean score of 44.46 (SD, 8.02) and 49.52 (SD, 8.08), respectively.

**Table 1:** Characteristics of the sample, stress, self-efficacy, and HRQOL subscales

Study variable	All (n = 696) (mean/SD)	Girls (n = 400) (mean/SD)	Boys (n = 296) (mean/SD)
Stress	0.29 (0.15)	0.33 (0.16)	0.24 (0.14)
Self-efficacy	31.08 (4.31)	30.25 (4.27)	32.21 (4.13)
Kidscreen			
Physical well-being	47.08 (9.34)	45.20 (8.66)	49.62 (9.62)
Psychological well-being	46.61 (8.42)	44.46 (8.02)	49.52 (8.08)
Autonomy and parents	52.60 (8.76)	51.97 (8.47)	53.46 (9.07)
Social support and peers	48.44 (8.47)	48.02 (8.42)	49.01 (8.51)
School environment	48.04 (8.57)	46.86 (7.97)	49.64 (9.10)

### Associations between stress, HRQOL subscales, and GSE

Stress was negatively and statistically significantly associated with all HRQOL subscales and GSE (Table 2, all *p* < 0.01): physical well-being (B =  − 25.60), psychological well-being (B =  − 38.43), autonomy and parents (B =  − 28.84), social support and peers (B =  − 21.05), school environment (B =  − 30.28), and GSE (B =  − 12.97).

**Table 2:** Linear regressions of stress (independent variable) on the HRQOL subscales (dependent variables) and GSE (dependent variable)

Study variable	B	95% CI	*p-*value
Physical well-being	− 25.60	− 29.57 to − 21.64	< 0.01
Psychological well-being	− 38.43	− 41.19 to − 35.67	< 0.01
Autonomy and parents	− 28.84	− 32.37 to − 25.30	< 0.01
Social support and peers	− 21.05	− 24.72 to − 17.37	< 0.01
School environment	− 30.28	− 33.63 to − 26.92	< 0.01
GSE	− 12.97	− 14.76 to − 11.18	< 0.01

In Table 3, findings from the linear regressions of GSE on the HRQOL subscales are listed. GSE was positively associated with all respective HRQOL subscales. The HRQOL subscale social support and peers revealed the largest regression coefficient (B = 2.31).

**Table 3:** Linear regressions of GSE (independent variable) on the HRQOL subscales (dependent variables)

Study variable	B	95% CI	*p*-value
Physical well-being	2.17	2.02 to 2.32	< 0.01
Psychological well-being	1.85	1.70 to 2.01	< 0.01
Autonomy and parents	2.12	1.94 to 2.30	< 0.01
Social support and peers	2.31	2.13 to 2.49	< 0.01
School environment	1.92	1.76 to 2.01	< 0.01

### Mediation effect of self-efficacy on the relationship between stress and HRQOL subscales

**Fig. 2:**
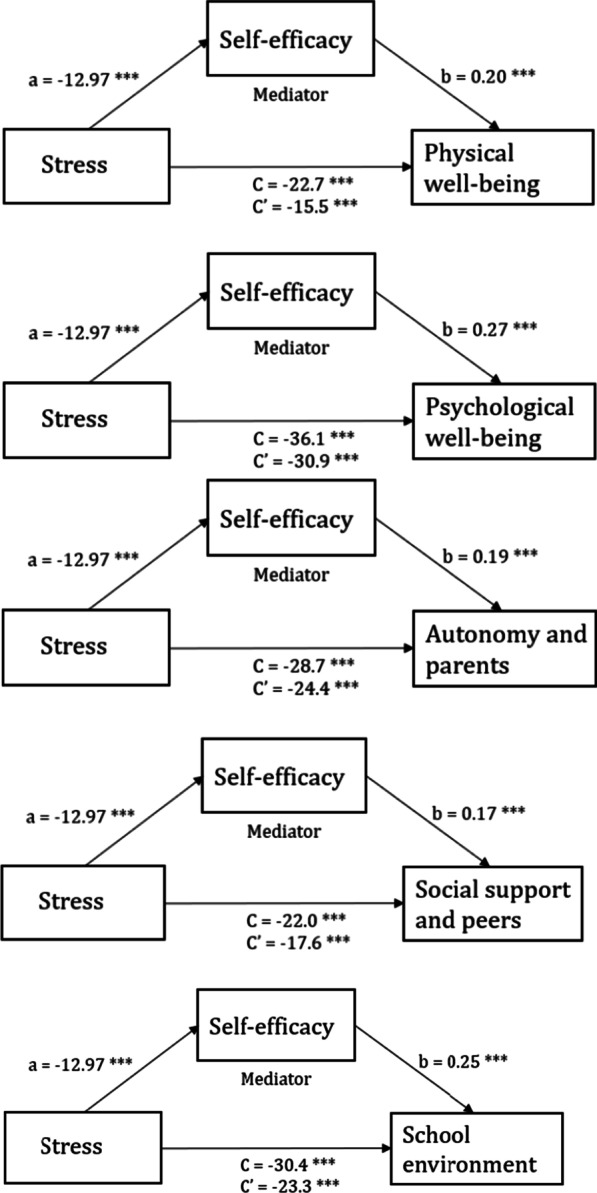
Mediation effect of self-efficacy on the association between stress and HRQOL subscales (physical well-being, psychological well-being, autonomy and parents, social support and peers, and school environment). *p < 0.05, **p < 0.01, ***p < 0.001

The mediation effect was assessed using the PROCESS macro [41]. After controlling for gender, ethnicity, parental marital status, and living conditions, a significant indirect effect was found for all selected HRQOL subscales (Fig. 2): physical well-being (95% CI [− 9.77 to − 4.81]), psychological well-being (95% CI [− 6.95 to − 3.75]), autonomy and parents (95% CI [− 6.45 to − 2.28]), social support and peers (95% CI [− 6.56 to − 2.44]), and school environment (95% CI [− 9.11 to − 5.08]).

Approximately one-fifth of the relationship between stress and HRQOL was explained by the mediating variable GSE. Our data revealed the lowest indirect effect (14.4%) for the HRQOL subscale psychological well-being, where the direct path of stress (C′ =  − 30.9) explained the majority of the total path (C =  − 36.1). The highest indirect effect (31.7%) was found for the HRQOL subscale physical well-being (Table 4). The estimates of regression coefficients are illustrated in Fig. 2.

**Table 4:** Direct and indirect effects presented as proportions (percentages) of the total effect controlled for gender, ethnicity, parental status, and living conditions

HRQOL subscale	Direct effect (%)	Indirect effect (%)
Physical well-being	68.3	31.7
Psychological well-being	85.6	14.4
Autonomy and parents	85.0	15.0
Social support and peers	80.0	20.0
School environment	76.6	23.4

## Discussion

This study evaluated the stress, HRQOL, and GSE in a sample from a school-based population of Norwegian adolescents and explored the associations between stress and HRQOL by modeling GSE as a possible mediator. As hypothesized, stress was negatively associated with all HRQOL subscales and self-efficacy. Furthermore, our findings revealed an indirect effect of self-efficacy on the relationship between stress and all selected HRQOL subscales after controlling for possible confounders. The highest indirect effect of self-efficacy, which explained approximately one-third of the reduction in the association between stress and HRQOL, was observed for the subscale physical well-being.

Our descriptive findings revealed overall low levels of perceived stress in a school-based sample of Norwegian adolescents, which is an important finding because stress, especially high levels of stress, is known to negatively impact the adolescents’ everyday life [13, 14, 16]. Therefore, we believe that it is important to differentiate between common everyday struggles and various perceived stress levels causing maladaptive behaviors. Inconsistencies in findings of stress levels in adolescents might partly be explained by methodological assessments, such as differences in scoring, measurements, and cutoff levels, used to categorize the instruments. Because adolescence is a transition period, which naturally evokes worries and struggles [1], the high levels of everyday struggles reported in Norwegian school-based samples of adolescents appear to be rational [6, 10].

Our findings showed that girls experienced higher stress levels and reported both lower mean GSE and HRQOL scores than boys, which is in accordance with the literature [16, 40–42]. Gender differences in HRQOL are common for adolescents and are more pronounced in older than younger children [43]. Several studies reported a lower HRQOL and mean GSE for girls than boys in adolescence [25, 44, 45]. Interestingly, a Norwegian study revealed no significant difference between the decline in HRQOL for boys and girls during 3 years in high school [45]. Because the decline in HRQOL is comparable between the genders in late adolescence, this might imply that the gender difference increases most rapidly from early adolescence. Thus, further understanding of the underlying mechanisms and associations of HRQOL in this population is important.

Although our findings revealed low levels of perceived stress, a negative association was observed between stress and all HRQOL subscales and self-efficacy, wherein the strongest associations were found for the subscale psychological well-being. Nevertheless, the directions of predicative assumptions should be addressed because stress and psychological well-being might interact in the adolescent’s everyday life, as some individuals might experience a reduction in psychological well-being due to the experience of stress and demands that exceed their resources. Other individuals might experience higher levels of stress due to concerns regarding their psychological well-being. This implies that stress may probably act as a cause (predictor) and/or a symptom (outcome) in relation to several aspects of the adolescents’ lives, which illustrates the complexity of stress. Another interesting finding was the statistically significant association between self-efficacy and the respective HRQOL subscales, revealing GSE as a predictor for HRQOL. Although HRQOL is defined as a multidimensional concept, the generic measure of self-efficacy (GSE) appears from an isolated perspective to have great potential for targeted intervention strategies for all dimensions of the HRQOL.

Our overall findings indicated that approximately one-fifth of the association between stress and the HRQOL subscales was explained by the indirect effect of self-efficacy when controlled for possible confounders. Hence, strategic interventions aimed at increasing the HRQOL often focus on strengthening self-efficacy because it is shown to be an important determinant of HRQOL. However, because stress is assumed to be a predictive factor for HRQOL, the lowest indirect effect of GSE was found for the subscale psychological well-being. These findings indicated that the degree of self-efficacy may not have the potential to influence psychological well-being in adolescents as expected, because stress itself appears to be the primary predictor. The highest indirect effect, which explained approximately one-third of the reduction in the association between stress and HRQOL, was found for the subscale physical well-being, which is especially interesting. It is important to highlight that the HRQOL subscale physical well-being does not represent physical activity levels but is theorized as the subjective perception of the physical well-being of the person’s own beliefs and expectations [21]. Despite this, previous findings suggested that self-perception of physical activity and fitness is associated with lower psychosomatic health symptoms in adolescents [46]. Because self-efficacy is defined as the confidence in the ability to perform a particular task, it appears logical that the adolescents probably perceived a decline in their physical well-being when their stress levels increased. Moreover, previous findings in a school-based population of Norwegian adolescents with persistent pain, revealed that self-efficacy mediated the relationship between pain intensity and HRQOL, herein the largest indirect effect of 67.2% was estimated for the HRQOL subscale physical well-being [25]. Hence, it appears that several reasons and different stressors, such as pain intensity and stress make maintaining high beliefs of their own capacity a challenge for Norwegian adolescents in a school-based population, which seems to be increasing the barriers for their physical well-being.

### Strengths and limitations

The mediation model in this study was built on assumptions of the current understanding of the research area. The assumptions and understandings were based on the empiric and research evidence provided by researchers within the field and aimed to provide new insights into the potential mechanisms underlying the relationship between perceived stress and HRQOL. Hence, we may only assume the direction of direct and indirect effects. Furthermore, because we only used cross-sectional data, we can only present associations revealed using our analyses and present evidence that confirmed our anticipated mediation model. However, no causal relationships could be identified. The mediation analysis was conducted using available and relevant possible confounders, such as gender and ethnicity. We did not have information regarding the parents’ education and/or salary indicating their socioeconomic status, which would be a more suitable covariate than parental status and living conditions. Thus, this should be considered a limitation of the present study. Another limitation is linked to the low overall response rate (41.8%) and the high variability of response rates across schools (from 92.1 to 8.6%). The response rate was lowest in two large schools in the east of Norway and highest in two small schools in the south of Norway, but due to General Data Protection Regulation laws, we could not collect information to assess whether the participants and nonparticipants nor the schools differed in any respect. However, in our sample, most adolescents had a parent with high education level and relatively high household income, which indicate that our findings may not be representative of Norwegian adolescents with a lower socioeconomic status. Our findings may only be generalized to a Norwegian school-based population of adolescents with a higher socioeconomic status. A clear strength of this study is that it had a comparatively large sample size in a research field where large studies are scarce. Because large study samples statistically affect the *p*-value, the present study provided clear estimates for clinical interpretation. Finally, the KIDSCREEN-27 questionnaire employed in this study used a 1-week recall period, which is considered an advantage for preventing recall bias [28, 47].

### Clinical implications

Because self-efficacy is regarded as a self-regulatory mechanism that is possible to change, a natural intervention strategy from a clinical perspective should be the enhancement of self-efficacy when aiming to improve HRQOL in a school-based population of adolescents. However, our findings indicated that stress itself explained the majority of the reduction in its association with all HRQOL subscales, especially in the subscale of psychological well-being, and thus, the corresponding lowest indirect effect of GSE. Based on these findings, it is reasonable to assume that intervention strategies that solely focus on self-efficacy among school-based adolescents experiencing stress might have a reduced potential for improvement in HRQOL. For instance, for adolescents with persistent pain, wherein the alleviation of pain itself might be a challenge and thus, in that case, self-efficacy is a more appropriate intervention target to increase HRQOL than pain. Therefore, this leads to the question of how to optimize intervention strategies targeting stress itself. Moreover, health care personnel, staff, and teachers in schools should be aware of the negative impact stress appears to have on the HRQOL in a school-based population of adolescents.

## Conclusions

Our findings extend prior research on the mechanisms underlying the relationship between perceived stress and HRQOL. Overall, the present study showed how important perceived stress is for adolescents with regard to all aspects of their HRQOL. As hypothesized, stress was negatively associated with all HRQOL subscales, and self-efficacy mediated these associations. Moreover, our findings revealed that perceived stress explained the majority of the reduction in HRQOL after testing for the mediating variable self-efficacy. Hence, for this study sample, stress itself appears to be an important target for future intervention to enhance HRQOL. Thus, such interventions should not purely focus on increasing self-efficacy to enhance HRQOL in adolescents. Our findings highlight the importance of a better understanding of the underlying mechanisms in adolescence to develop more strategic and accurate interventions.

## Data Availability

The datasets used and/or analyzed during the present study are not publicly available due to the General Data Protection Regulation laws but are available from the corresponding author on reasonable request and with permission from the Norwegian Centre for Research Data.

## Abbreviations

CI: Confidence interval
GSE: General self-efficacy
HRQOL: Health-related quality of life
PSQ: Perceived Stress Questionnaire
SD: Standard deviation
